# Cytogenetic damage analysis in mice chronically exposed to low-dose internal tritium beta-particle radiation

**DOI:** 10.18632/oncotarget.25282

**Published:** 2018-06-08

**Authors:** Sandrine Roch-Lefèvre, Eric Grégoire, Cécile Martin-Bodiot, Matthew Flegal, Amélie Fréneau, Melinda Blimkie, Laura Bannister, Heather Wyatt, Joan-Francesc Barquinero, Laurence Roy, Mohamed Benadjaoud, Nick Priest, Jean-René Jourdain, Dmitry Klokov

**Affiliations:** ^1^ Institut de Radioprotection et de Sûreté Nucléaire, IRSN, Pôle Santé et Environnement, Direction de la Santé, Fontenay-aux-Roses, France; ^2^ Radiobiology and Health, Canadian Nuclear Laboratories, Chalk River, Ontario, Canada; ^3^ Institut de Radioprotection et de Sûreté Nucléaire, IRSN, Direction des Affaires Internationales, Fontenay-aux-Roses, France; ^4^ Department of Biochemistrty, Microbiology and Immunology, University of Ottawa, Ottawa, Ontario, Canada; ^5^ Present address at: Autonomous University of Barcelona, Faculty of Biosciences, Cerdanyola del Vallès, Spain

**Keywords:** genotoxicity, chronicle, low-dose, tritium exposure

## Abstract

The aim of this study was to carry out a comprehensive examination of potential genotoxic effects of low doses of tritium delivered chronically to mice and to compare these effects to the ones resulting from equivalent doses of gamma-irradiation. Mice were chronically exposed for one or eight months to either tritiated water (HTO) or organically bound tritium (OBT) in drinking water at concentrations of 10 kBq/L, 1 MBq/L or 20 MBq/L. Dose rates of internal β-particle resulting from such tritium treatments were calculated and matching external gamma-exposures were carried out. We measured cytogenetic damage in bone marrow and in peripheral blood lymphocytes (PBLs) and the cumulative tritium doses (0.009 – 181 mGy) were used to evaluate the dose-response of OBT in PBLs, as well as its relative biological effectiveness (RBE). Neither tritium, nor gamma exposures produced genotoxic effects in bone marrow. However, significant increases in chromosome damage rates in PBLs were found as a result of chronic OBT exposures at 1 and 20 M Bq/L, but not at 10 kBq/L. When compared to an external acute gamma-exposure *ex vivo*, the RBE of OBT for chromosome aberrations induction was evaluated to be significantly higher than 1 at cumulative tritium doses below 10 mGy. Although found non-existent at 10 kBq/L (the WHO limit), the genotoxic potential of low doses of tritium (>10 kBq/L), mainly OBT, may be higher than currently assumed.

## INTRODUCTION

Tritium (^3^H), a radioactive isotope of hydrogen, is a byproduct of the nuclear industry released into the environment and it represents a significant public concern for potential health effects [1–3]. These concerns have been on a rise lately due to the expected growth of nuclear power production world-wide, the development of nuclear fusion technology, and the continuing uncertainties related to potential health effects of tritium [3, 4]. The uncertainties stem from a wide range of relative biological effectiveness (RBE) values measured experimentally for tritium [5–9]. The RBE value shows how effective a particular type of radiation is at causing detrimental biological effects relative to a reference photon radiation, such as γ− or X-rays. Based on a review of RBE and other information available, the International Commission for Radiological Protection (ICRP) currently recommends in calculating effective dose, a radiation weighting factor of 1 for all low Linear Energy Transfer (LET) radiations, including β-particles that tritium emits [10, 11]. However, theoretical considerations of track structure and other aspects of low-energy *β*-radiation, as well as experimental evidence that the RBE for tritium may be more than twice that of other low LET radiations, indicate that low-energy *β*-emitters may have greater biological effectiveness per unit absorbed dose and as such, an RBE of 1 for tritium may not be appropriate (reviewed in [12]). From a radiation protection point of view, such uncertainties in evaluating effective dose for tritium may lead to uncertainties in its guidance levels. Indeed, the current guidelines in situations of prolonged radiation exposure of the public are based on the approach proposed by the ICRP [13]. According to the ICRP, in existing exposure situations, it is prudent to restrict the prolonged component of the individual dose to 0.1 mSv from 1 year's consumption of drinking-water [14]. In fact, there are remarkable differences in the drinking water tritium standards between countries (e.g. 100 Bq/L in most of countries of the European Union, 7,000 Bq/L in Canada, 7,700 Bq/L in Russia, 30,000 Bq/L in Finland and more than 76,000 Bq/l in Australia) [15].

Tritium transmutates to a stable isotope of helium through *β*-particle decay, emitting a low-energy electron with a mean energy of 5.7 keV along with an anti-neutrino particle. The path length of a *β*-particle emitted by tritium is only 0.6 μm, which is smaller than a cell nucleus diameter [9]. Therefore, tritium only poses a health risk as an internal hazard if ingested through drinking water or food, or inhaled or absorbed through the skin. As an isotope of hydrogen, tritium readily forms water molecules, and consequently is very mobile in the environment, and incorporates into organic molecules to form organically bound tritium (OBT) [4]. Tritium mainly exists in the environment as tritiated water (HTO) or OBT.

Biochemically, HTO behaves like water in the body, equilibrating throughout the fluid compartments [16]. A small fraction of HTO may also exchange with hydrogen atoms and become incorporated into organic molecules as OBT [7, 9]. Unlike HTO, OBT uptake is expected to be heterogeneously distributed in cells and tissues [7, 9]. OBT in the body can be incorporated into organic molecules (amino acids, sugars, proteins, etc.) that have slower turnover rates and this may result in a higher absorbed dose. Furthermore, tritium as OBT may be incorporated into genomic DNA causing highly localized damage to genetic material [7, 9, 17]. Such differences in HTO and OBT biochemical characteristics and the inability to easily trace two tritium forms and determine their cellular and sub-cellular distribution greatly contribute to the uncertainties related to biological effectiveness of tritium *β*-particle irradiation.

RBE value, being an experimentally determined parameter, may vary depending on cell types and end-points used. Historically, cell inactivation or cell killing has long been the gold standard for RBE measurements [18, 19]. However, for the purpose of radiological protection, which mostly seeks to evaluate risks of cancer resulting from low-dose exposures, cell killing plays a secondary role to cancer driving changes and mechanisms. To this end, chromosome aberrations have been widely used to measure RBE of various types of radiation [20, 21].

Theoretical and experimental studies have shown tritium *β*-particles to be particularly efficient in producing double-strand breaks (DSBs) in DNA, including complex DSBs [22–25] that are considered to be the most deleterious type of DNA damage which, if left unrepaired or improperly repaired, may lead to chromosome damage, genomic instability and cancer [26–28]. *In vivo* studies, which are most relevant to human radiological protection, aiming at evaluating the RBE of tritium have also been carried out (reviewed in [12]). However, the heterogeneous design of most studies, in particular the use of high doses and dose rates, the choice of reference radiation type (X-ray vs. γ-radiation) and relating tritium *β*-radiation to γ-radiation effects obtained at different dose rates globally lead to resulting tritium RBE values that are difficult to interpret. UNSCEAR provides a recent review on tritium including RBE [29]. The committee concludes that RBE values derived from about 50 *in vivo* and *in vitro* experiments on mammals, for different end points, ranged from 1.0 to 5.0 (centered around 2–2.5) and from 0.4 to 8.0 (centered around 1.5–2) with regard to gamma rays and orthovoltage X-rays, respectively. Studies also showed a general tendency of RBE values to increase with lower doses. Moreover, this independent review of the scientific literature on the radiobiological effects of tritium exposure shows that most experimental studies were performed 20 to 30 years ago. While this work was competently performed at the time, it did not use procedures developed more recently that are often more sensitive and can use multiple approaches. The application of more recent techniques, including DNA damage analyses, would be helpful in reinvestigating aspects of tritium dosimetry and effects.

To address these issues, an international collaboration between the Institute for Radiological Protection and Nuclear Safety (IRSN, France) and Canadian Nuclear Laboratories (CNL, Canada) was established to conduct a large scale animal *in vivo* study aiming at assessing comprehensively the biokinetics and the potential biological effects of chronic low-dose tritium exposures. The end-points used in this large scale study included biomarkers of physiological health status, inflammation, cytogenetic damages, DNA damage and repair, as well as systemic end-points, such as life-span and cancer rates. Here, we report the data on cytogenetic toxicity of chronic (1 and 8 months) exposure to occupationally relevant tritium concentrations (10 kBq/L, 1 MBq/L and 20 MBq/L) in drinking water in the form of both HTO and OBT. This study included a reference external γ-radiation exposure at dose rates equivalent to the one resulting from 1 and 20 MBq/L tritium exposures. We present our assessment of the dose-response relationship as well as the RBE for *β*-particle irradiation from OBT consumed with drinking water.

## RESULTS

### Bone marrow MN assay

**Figure 1:**
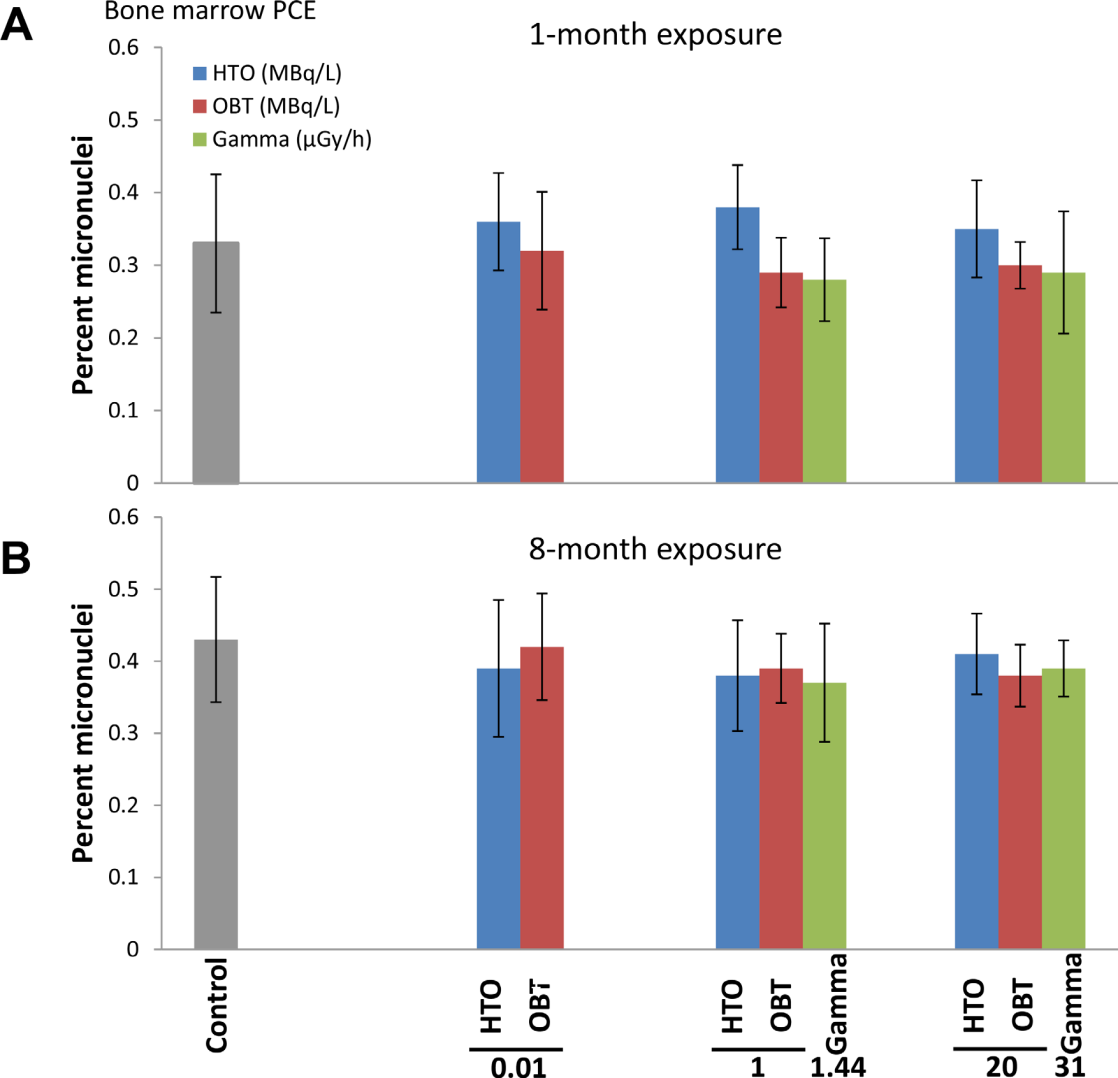
Frequencies of micronucleated PCE (MN-PCE) in bone marrow of mice treated *in vivo* with indicated doses of HTO or OBT in drinking water or γ-radiation for 1 (**A**) or 8 (**B**) months.

Cytogenetic damage in bone marrow cells was measured using the micronucleus test, a widely accepted technique for measuring genotoxic effects of various agents and environmental conditions (Maier and Schmid 1976; Hayashi *et al.* 1994). The frequency of polychromatic erythrocytes containing micronuclei (mn-PCE) in control mice sham-irradiated for 1 month was 0.33% (Figure 1A). One-month treatment of mice with 10 kBq/L, 1 or 20 MBq/L of tritium in the drinking water as HTO or OBT did not lead to statistically significant changes in the frequencies of mn-PCE. Similarly, one-month γ-irradiation did not result in a significant change in the frequencies of bone marrow mn-PCE.

In the 8-month treatment experiment, the frequency of bone marrow mn-PCE in the control cohort was expectedly higher than in the 1-month control animals (Figure 1B), reflecting accumulation of cytogenetic abnormalities with age [30]. Treatment of mice with tritium in drinking water for 8 months, both as HTO and OBT, did not lead to increases in the frequencies of mn-PCE. Similar to the 1-month exposure, 8-month γ-irradiation did not produce higher rates of bone marrow mn-PCE. This data suggests that chronic exposure of mice to low doses of internal β-irradiation from HTO or OBT in drinking water at concentrations of 10 kBq/L, 1 or 20 MBq/L, as well as to external γ-irradiation at dose rates of 1.44 and 31 μGy/h, does not affect the level of cytogenetic damage in bone marrow cells.

Proliferation/maturation index in the bone marrow tissue was assessed by measuring PCE/NCE ratio (Table 1). This parameter is indicative of either bone marrow cytotoxicity or any other perturbations in the process of erythropoiesis. We failed to observe statistically significant changes in the PCE/NCE cell ratios (Table 1) by any of the irradiation types or treatment lengths. This data suggests that the mouse *in vivo* irradiation conditions used in this study did not affect the bone marrow erythropoiesis or cytotoxicity. Additionally, unaffected PCE/NCE ratios confirm the lack of a bias in the data for bone marrow mn-PCE [31].

**Table 1:** Bone marrow PCE/NCE ratios in mice exposed to low doses of tritium in drinking water and to external γ-irradiation for 1 or 8 months

Treatment group	No of mice	PCE/NCE^*^	PCE/NCE S.E.M
1 month			
Control	10	1.08	0.10
HTO 10 kBq/L	10	1.12	0.08
HTO 1 MBq/L	10	0.98	0.07
HTO 20 MBq/L	10	1.05	0.08
OBT 10 kBq/L	10	1.04	0.06
OBT 1 MBq/L	10	1.11	0.09
OBT 20 MBq/L	10	1.07	0.08
Gamma 1.44 μGy/h	12	1.02	0.05
Gamma 31.0 μGy/h	12	1.04	0.06
8 months			
Control	10	0.97	0.12
HTO 10 kBq/L	9	1.01	0.09
HTO 1 MBq/L	12	0.97	0.09
HTO 20 MBq/L	12	0.93	0.07
OBT 10 kBq/L	10	1.11	0.11
OBT 1 MBq/L	10	0.99	0.09
OBT 20 MBq/L	10	1.01	0.12
Gamma 1.44 μGy/h	10	0.97	0.07
Gamma 31.0 μGy/h	10	0.96	0.09

^*^None of the changes in treatment groups were statistically significant compared to corresponding control (Student's *t*-test).

### Blood Lymphocytes M-FISH Analysis

Frequencies of chromosome aberrations are often measured to assess the genotoxic and carcinogenic potential of a substance or environmental condition [32–35]. Here, we used the M-FISH technique that utilizes chromosome-specific florescence probes to quantify, in each PBL metaphase, both unstable (e.g. dicentrics and centric rings) and stable (e.g. translocations and insertions) aberrations as well as chromosomes fragments, leading to an evaluation of a global chromosome damage rate by converting the observed aberrations into chromosome breakpoints. In fact, this allowed us to achieve a satisfactory degree of statistical confidence by increasing the number of scored radiation-induced events while scoring a usual number of cells as previously demonstrated [36].

**Figure 2:**
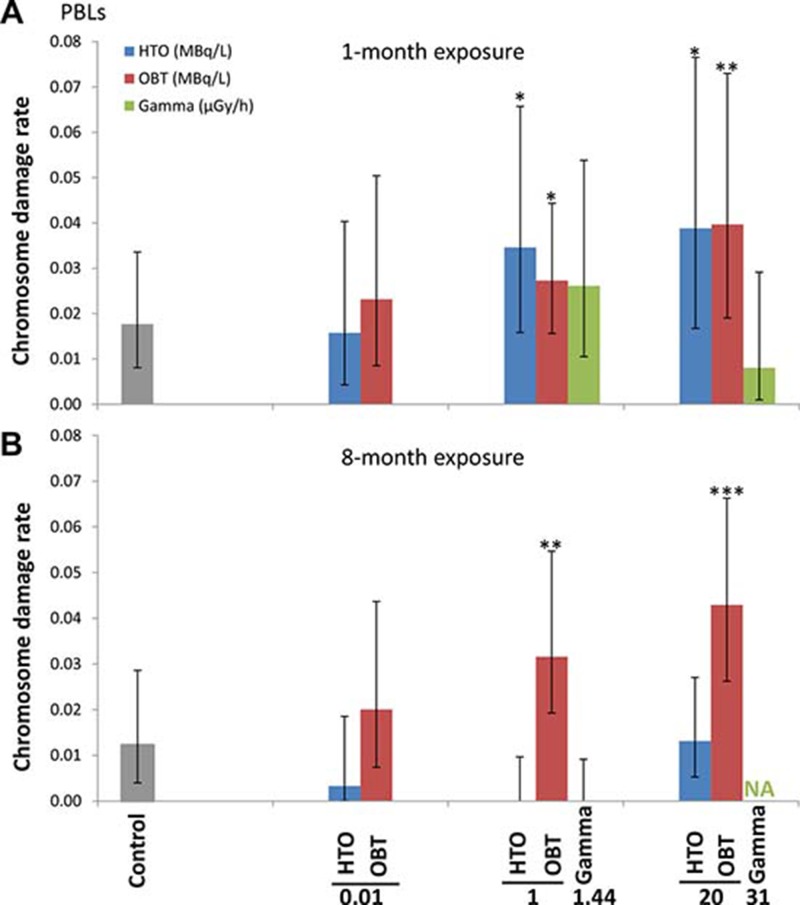
Chromosome damage rates measured using M-FISH in peripheral blood lymphocytes of mice treated *in vivo* with indicated doses of HTO or OBT in drinking water or γ-radiation for 1 (**A**) or 8 (**B**) months.

Results of the one-month exposure experiment are presented in Figure 2A. With exposure to 10 kBq/L tritium, both HTO and OBT were found not to affect the chromosome damage rates. However, a statistically significant increase in chromosome damage was observed when mice were treated with HTO and OBT at higher concentrations (1 and 20 MBq/L). γ-irradiation of mice for one month at either 1.44 or 31 μGy/h did not affect the level of chromosome damage in blood lymphocytes. These two γ-radiation dose rates correspond to an internal *β*-particle dose-rate in mice receiving 1 and 20 MBq/L tritium in drinking water, respectively (Table 2).

**Table 2:** Estimated dose rates and total cumulative doses resulting from the 1 and 8-month exposures of mice with low doses of tritium in drinking water or with external gamma-irradiation

Treatment group	Dose rate (μGy/h)	Cumulative dose for 1 month	Cumulative dose for 8 months
		(mGy)	(mGy)
Control	0	0	0
HTO 10 kBq/L	0.015	0.009	0.083
HTO 1 MBq/L	1.54	0.9	8.3
HTO 20 MBq/L	30.9	18.3	166
OBT 10 kBq/L	0.016	0.008	0.088
OBT 1 MBq/L	1.66	0.97	8.95
OBT 20 MBq/L	33.7	21.7	181.3
Gamma 1.44 μGy/h	1.44	0.96	7.7
Gamma 31 μGy/h	31.0	20.8	166

Extending the length of exposure to 8 months did not result in greater chromosome damage rates in 10 kBq/L-tritium treatment groups (HTO and OBT) compared with the sham-irradiated control (Figure 2B). Interestingly, 8-month exposure to HTO at 1 and 20 MBq/L, unlike 1-month exposure, did not result in increased rates of chromosome damage. For OBT treatment at 1 and 20 MBq/L, we found statistically significant 2- and 3-fold higher rates of chromosome damage (*p* < 0.01 and *p* < 0.001, respectively). Noteworthy, chromosome damage rates were somewhat higher for 20 vs. 1 MBq/L for both 1- and 8-month OBT exposure lengths (Figure 2A and 2B). Similarly, no excess damage was observed for γ-irradiated animals (due to technical issues only 1.44 μGy/h samples were available for analysis).

**Figure 3:**
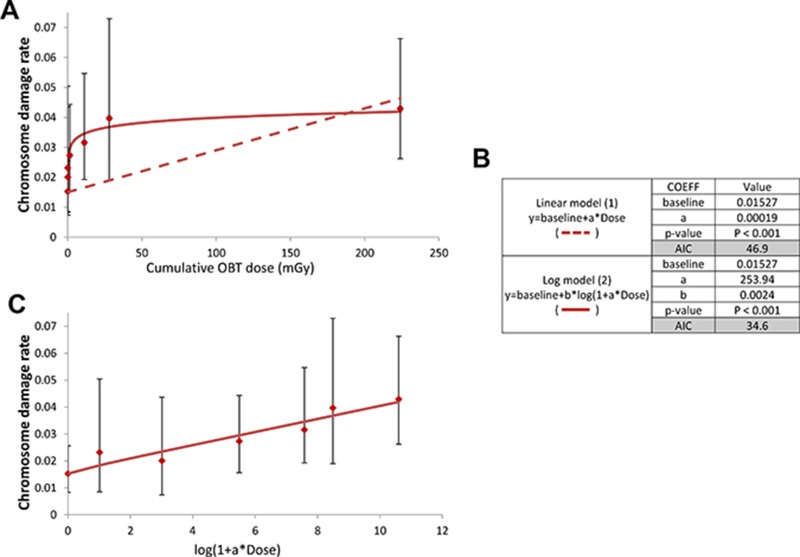
Dose-response for chromosome damages induced by *in vivo* chronic OBT exposure from both 1- and 8-month experiments (**A**) chromosome damage rates resulting from chronic OBT exposure were plotted as a function of OBT-cumulative dose. The dotted line represents the curve fitted using a linear model (1). The full line represents the curve fitted using a logarithmic model (2). Error bars represent the upper and lower 95% confidence intervals assuming Poisson distribution. (**B**) Table summarizing the coefficients, *p*-value and AIC associated with each model (**C**), dose-response for OBT-induced chromosome damage that illustrates dose-dependence. Chromosome damage rates were plotted as a function of OBT-cumulative dose rescaled according to the logarithmic term in the equation (2). Error bars represent the upper and lower 95% confidence intervals assuming Poisson distribution.

**Figure 4:**
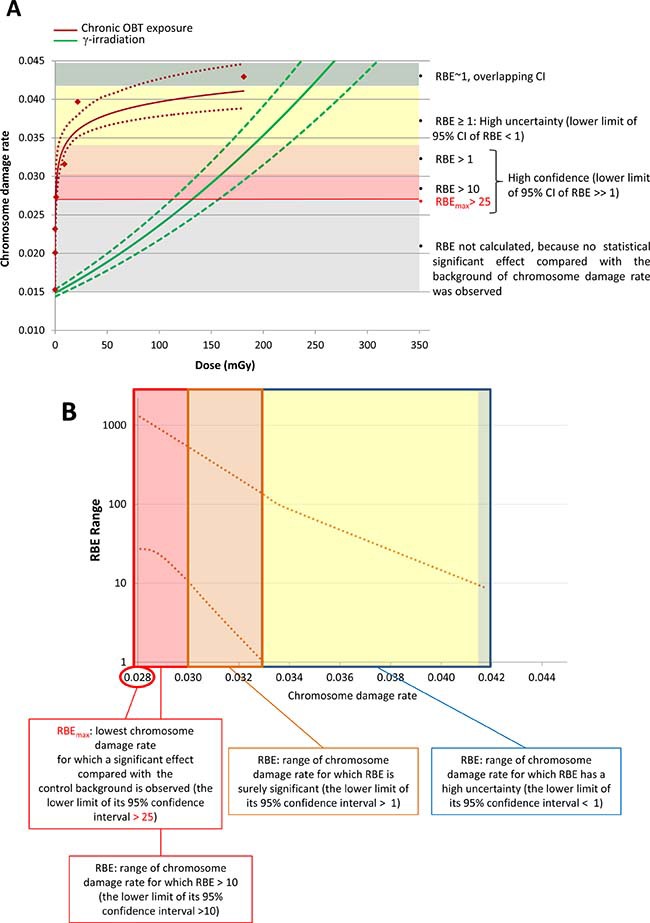
RBE estimation of OBT with respect to chromosome damage rate induced in mouse PBLs Color coded areas correspond to areas with different RBE estimates made using the shown dose-response fits. (**A**) Dose-response fits of chromosome damage rate in mouse PBLs upon chronic *in vivo* OBT or acute *ex vivo* γ-exposures. Upper and lower dotted lines show the 95% confidence intervals of the fitted dose-responses. Legend on the right describes RBE estimates of each of the color coded areas. (**B**) Upper and lower curves representing the 95% confidence intervals of the RBE derived from data in a.

To further examine dose-dependence of the chromosome damage level in PBLs, the rates of chromosome damage induced after OBT exposure were plotted against the total cumulative doses from both 1- and 8-month experiments (dose estimates are given in Table 2). Dose-response curves were then constructed using two different models, either a linear model (1) (Figure 3A, dotted line) or a logarithmic model (2) (Figure 3A, solid line). Significant dose-effect correlations were observed for both models (*p* < 0.001, Figure 3B). When the Akaike information criteria (AIC) values were compared, we observed that the goodness of fit of the logarithmic model (2) was significantly better relative to the linear model (1) (Figure 3B, AIC = 34.6 and 46.8, respectively). To visually show such goodness of fit of the logarithmic model, the data was presented in a logarithmic dose scale that displays a dose-dependent increase in the level of chromosome damage (Figure 3C). Neither HTO nor chronic external γ-irradiation resulted in such correlation with dose. For γ-irradiation however, only a limited number of data points were available. This was due to the inability to achieve the dose rate corresponding to 10 kBq/L-tritium exposure (0.016 μGy/h) and the loss of samples from the 31 μGy/h 8-month group. Furthermore, as no biological effect (*i.e.* no significant excess of chromosome damage) was observed after chronic external γ-irradiation, the calculation of RBE for tritium was technically unachievable using this data as a reference. Indeed, RBE is defined as the ratio of the absorbed dose of a reference radiation to the absorbed dose of the radiation that is required under similar conditions to produce an identical level of biological response. To overcome this technical issue, we used a dose-response curve for chromosome damage rates previously generated using mouse PBLs irradiated *ex vivo* with acute γ-irradiation [36]. This data fit with a linear-quadratic regression (shown in Figure 4A) was used as a reference for estimating the ranges of RBE values for OBT. The different estimated RBE ranges are color coded in Figure 4 and provided as text next to the plot area. The highest range of RBE estimate (10 < RBE < 25) was mapped to the area of significant but moderate chromosome damage increases (Figure 4B, red area) which correspond to cumulative doses of OBT lower than 1 mGy (Figure 4A, red area). As chromosome damage rate increased with the increase of exposure dose, the RBE values rapidly decreased and reached the value of 1 at doses >200 mGy (Figure 4).

## DISCUSSION

Biological effects of *β*-radiation from tritium are typically considered in the context of radiological protection, for both nuclear workers occupationally exposed to tritium and for public. Therefore, experimental studies that aim to determine the RBE of tritium need to focus on the low-dose range, wherein deviation from linearity in dose-responses has been demonstrated in a great number of studies (reviewed in [37] and [38]). An additional factor important to keep in mind while studying the biological effects of tritium is the preference of chronic exposures vs. acute ones. Unlike many previous studies, this study attempted to reproduce these requirements of very low-dose and dose-rate irradiation conditions. Additionally, the two major types of tritium, HTO and OBT were also included, as their different biochemical properties defining their intra-cellular localization and biokinetics suggest that they may produce biological effects with different efficiencies [39].

First examined, was whether cytogenetic damage in mouse bone marrow erythroblasts can be induced by HTO, OBT, or equivalent ^60^Co γ-radiation. Bone marrow is one of the most radiosensitive tissues because of active processes of haematopoiesis and lymphopoiesis [40]. The route of treatment was *via* drinking water since it mimics the most natural way of human exposure to tritium, unlike intraperitoneal injection used in many previous studies. Neither one nor eight months of such treatment were able to produce statistically significant excesses of micronucleated PCE in bone marrow. These cells containing micronuclei are formed as a result of chromosome breaks or lost chromosomes during the last mitosis of erythroblasts and are commonly used to evaluate genotoxicity *in vivo* [41]. Ruled out, was the possibility that damaged erythroblast cells had been selectively eliminated from entering the erythrocyte population and thus causing underestimated scores of mn-PCE by quantifying PCE/NCE ratios. The lack of bone marrow cytotoxicity is consistent with our previous data, generated from the same mice used in this study, showing the lack of apoptosis in the spleen after 1 month of HTO treatment at all three concentrations [42]. The bone marrow data that we obtained in mice suggest that HTO and OBT in drinking water at concentrations comparable to or exceeding those used in regulatory standards in some countries by 3–4 orders of magnitude do not exert cytotoxicity and genotoxicity in bone marrow erythroblasts. To our knowledge, no other studies have examined genotoxicity of such low doses and dose rates of tritium in mouse bone marrow *in vivo*. Kozlowski *et al.* [43] showed increased frequencies of stable chromosomal aberrations in bone marrow cells of mice treated with ~1 GBq/L HTO during pregnancy and in their offspring. However, the dose delivered to mice in that study was ~50-fold higher than the highest dose used in our study. Similarly, whole body external γ-irradiation at 1.44 μGy/h and 31 μGy/h for 1 or 8 months (total cumulative doses were 1 and 20 mGy, and 8 and 155 mGy, respectively) did not produce elevated rates of micronucleated bone marrow cells, consistent with previous findings [44].

Typically, experimental human studies of radiation effects are performed using peripheral blood lymphocytes irradiated *ex vivo* [45, 46]. It is also one of the first tissues that is used to assess cytogenetic effects resulting from accidental human exposures *in vivo* for population triage and biodosimetry purpose [47]. In this study, chromosome aberrations in peripheral blood lymphocytes were evaluated for mice chronically exposed to low doses of HTO, OBT and γ-radiation. Both HTO and OBT were found to induce increased levels of chromosome aberrations at concentrations of 1 and 20 MBq/L following a 1-month exposure. Excess damage was not observed for HTO when the exposure was protracted to 8 months. It is tempting to speculate that upon longer exposure to HTO compensatory repair mechanisms may be triggered. Consistent with this hypothesis, the level of cytogenetic damage at 8 months of HTO treatment was lower than that in the control (although not statistically significant, but observed for all three HTO concentrations). A similar “compensatory” effect was observed in a previous study for chronic *in vivo* γ-irradiated mouse blood leucocytes using DNA DSB as an end-point [48].

However, this presumably compensatory effect was not seen for OBT treated mice. Instead, chromosome aberrations increased with dose. To explain these differences between HTO and OBT, subcellular distribution and *β*-particle track characteristics may be considered. Indeed, tritium fixed as OBT in the proximity of or within chromatin will likely produce localized clustered DNA damage due to the low track length (0.56 μm in water) and relatively high ionization density of a *β*-particle. The clustered DNA damage is not readily repairable [49], unlike uniformly distributed DNA lesions which may be produced by HTO freely moving inside the nucleus or the cytoplasm. In fact, since tritium is a low-energy beta emitter, a cumulative dose is dependent on the volume in which the energy is deposited. Assuming that OBT accumulates only within the nucleus of the lymphocyte, the cumulative dose would be 2 orders of magnitude higher than the one resulting from a homogeneous tritium distribution (Aurélie DESBREE, personal communication). Obviously, even a greater dose increase would be expected if OBT accumulates preferentially in the chromatin. However, this reasoning is purely hypothetical and microdosimetry of OBT remains an issue that needs further investigation. It is consequently unclear whether any of the above scenarios took place and caused higher than expected cytogenetic effects of OBT observed in this study.

Constructing a dose-response is a classical and powerful means of characterizing a certain type of radiation in terms of its biological effects. In constructing dose-responses here, we brought together the data obtained for different concentrations of tritium (10 kBq/L, 1 MBq/L and 20 MBq/L) corresponding to three different dose rates of β-irradiation (0.016, 1.66 and 33.7 μGy/h, respectively - Table 2) which may seem questionable in terms of a bias due to a potential dose rate effect. However, at such low dose rates, the rate of ionizing tracks per cell per hour is so low (even at the highest dose rate it is estimated at 0.004 tracks per cell per hour) that the vast majority of chromosome breaks would be produced by a single track. In such case, no dose rate effects is expected [50] thus allowing us to combine the data for dose-response construction. The resulting dose-response for OBT showed that the effect increased with dose logarithmically, not linearly. HTO exposure in contrast did not result in such a dose-response: neither logarithmic nor linear relationship was observed. Interestingly, after the initial increase in the level of chromosome damage compared to the control at 1 month of exposure, a decrease was seen at 8 months of exposure. This may indicate that a compensatory mechanism that protects from further accumulation of chromosome damage past 1 month of HTO exposure was induced.

Failla and Henshaw first introduced the concept of relative biological effectiveness in 1931 [51]. Although conceptually simple by its definition [52], RBE cannot be uniquely determined for a given type of radiation since its calculation involves experimental results and includes experimental uncertainties with many variables, such as particle/photon energy/spectrum, dose, dose rate, cell or tissue type, and biological end-point [53]. For environmental and occupational radiological protection, the most relevant parameter is the low-dose limiting RBE. Since RBE can vary greatly with dose rate and radiation quality, an imperative for its accurate evaluation in the case of chronic exposures is the use of very low dose rates of equivalent γ-radiation exposures [9, 54]. Unfortunately, due to the lack of detectable effects, as well as the loss of samples, no dose-response curve for chromosome damage after chronic γ-irradiation could be generated. This makes calculation of RBE using chronic *in vivo* γ-radiation as a reference not possible. Instead, a dose-response curve for chromosome damage previously generated for acutely *ex vivo* γ-irradiated mouse blood lymphocytes was used as a reference in calculating RBE [36]. Indeed, using dose-response data produced *ex vivo* is a well-accepted way of evaluating effects of ionizing radiation [50]. It is also reasonable to expect that the use of such reference radiation data would not lead to overestimated RBE of OBT since it is well documented that chronic γ-irradiation induces, for the same absorbed dose, less chromosome damage compared to acute γ-irradiation [50].

Our RBE estimates indicated fairly high (>25) values for very low OBT doses (<1 mGy). However, as dose increased, RBE decreased to become non significantly higher than 1 for chromosome damage rate of 0.033, corresponding to cumulative OBT doses of >5 mGy. The RBE values for very low OBT doses produced in this study may appear unusually high. However, such dependence of RBE of particle radiations on dose, *i.e.* RBE increases as dose decreases, is a known phenomenon [20, 55] and these high RBE values were observed for moderate increases of chromosome damage corresponding to around 100 mGy of external γ-radiation (Figure 4). Moreover, it is not clear how such moderate increases in chromosome aberration rates may translate into the cancer risk; the latter was not accessed within this study. Other end-points have also been used in the literature for RBE evaluation purposes, including cell killing and DNA DSB rates [25, 56]. However, chromosome aberrations and cancer incidence remain the most relevant end-points for radiological protection. Several epidemiological human studies examined chromosome aberrations in peripheral blood lymphocytes and found positive correlations between the level of exposure to tritium and cytogenetic damage [57, 58]. Consistent with the data presented here, RBE values for tritium effects assessed using chromosome aberrations in human peripheral blood lymphocytes were reported to be higher than 1 (between 2 and 3) depending on the dose [20, 59]. We nevertheless observed higher RBE values for tritium effects compared with those from the literature; however, this is not surprising given the fact that the cumulative tritium doses used here (<200 mGy) have never been assessed before in terms of chromosome damage induction, and that, as a general rule, RBE increases as dose decreases.

In this study we observed different results for bone marrow cells and peripheral blood lymphocytes, suggesting that tritium effects are tissue specific. Although it is not clear what mechanisms could be responsible for such differences, they may be related to differences in lymphopoiesis vs. haematopoiesis, and in associated DNA metabolisms that define how tritium is exerting its effects on DNA. Consistent with our results, reduced efficiency of HTO in bone marrow cells compared with blood lymphocytes was reported for chromosome aberrations [60]. For mice, very low tritium RBE value of 0.5 in bone marrow was derived [12] using data from Kozlowski *et al.* [43].

Interestingly, at 10 kBq/L neither HTO nor OBT caused any chromosome damage, suggesting a threshold for the dose-effect relationship. It is also important to note that although 10 kBq/L leads to a very low cumulative dose (*i.e.* 0.09 mGy in 8 months, Table 2) it exceeds in fact 100 – 1000 times the typical levels of tritium in drinking water near nuclear facilities [61]. This fact limits the practical use of RBE for OBT values derived from our results, a note that is also true for other studies. The World Health Organization recommends 10 kBq/L as a standard limit [13]; however, many jurisdictions have suggested *de minims* guidelines or investigation/action levels for tritium management that are lower than the WHO standards [3]. The lack of genotoxic effects observed at 10 kBq/L in the present study shows that this value seems to be conservative as a standard limit. However, as long as the threshold lower limit of induction of chromosome damage is not precisely determined for chronicle OBT exposure, a caution principle may be applied.

## MATERIALS AND METHODS

### Mice

Adult male C57BL/6J mice (Jackson laboratory, Bar Harbor, ME, USA) aged 8 weeks were used in the study. The animals were acclimatized for a minimum of one week following their arrival and then randomly assigned to experimental groups (10 mice per group). All animals were housed and treated in the pathogen-free Biological Research Facility at CNL (Chalk River, ON, Canada). Mice were maintained in Thoren cages. Animals were fed *ad libitum* with Charles River Rodent Chow and their health status was examined daily. Constant temperature (23°C), stable, sufficient air ventilation, and 12-h light/dark cycle were maintained in the facility. Tests for pathogens were performed routinely and all tested mice were negative. All experimental animal protocols were approved by the local Animal Care Committee (Protocol no. 09–05) and were consistent with the *Guidelines of the Canadian Council on Animal Care* [62].

### Tritium exposures

For HTO exposures, tritiated water stock (3.7 × 10^9^ kBq/L) obtained locally from the National Research Universal reactor facility at Canadian Nuclear Laboratories was used. The stock was diluted in reverse osmosis animal drinking water to obtain 10 kBq/L, 1 MBq/L and 20 MBq/L working concentrations. For OBT exposures, ^3^H-labeled amino acids (Perkin Elmer; Alanine, 65–85 Ci/mmol; Proline, 25–55 Ci/mmol; Glycine, 30–60 Ci/mmol) were diluted in animal drinking water to produce 10 kBq/L, 1 MBq/L and 20 MBq/L working concentrations which were verified using Tri-Carb 1900 liquid scintillation counter (Packard Instruments, Downer's Grove, IL, USA). Animals were given the tritium containing water *via* bottles *ad libitum*, and it was replaced with freshly prepared water every two weeks. Control mice were maintained in a rack with positive air pressure to avoid tritium contamination through breathing air. Negative air pressure was maintained in the racks hosting mice exposed to tritium. The animals were subjected to HTO or OBT drinking water for 1 month (4 weeks) or 8 months (32 weeks).

### Dose rate calculations

Evaluation of internal β-particle dose rates resulting from the tritium treatments were derived from the data obtained in separate experiments designed to measure uptake and retention of tritium [63]. Tritium concentration in mouse tissues were measured at time points of 0, 1, 7, 15, 21 and 30 days and percentage (P) of the input tritium concentration in drinking water was calculated for each time period using trapezoid areas of the biokinetics curves. Then dose was evaluated using the following formula:

Xt = D × P × b × E × T,

where Xt is dose to mouse in Gy per a time period t, D is drinking water tritium concentration in Bq/L, P is percentage of tritium tissue concentration relative to that in drinking water over a time period t, b is mean energy of a b-particle and equals to 5.7 × 10–3 MeV, E is the conversion factor from MeV to Joules and equals to 1.6021 × 10–13 J/MeV, T is the number of seconds in a time period t. Water density was assumed to be 1 kg/L. For 1-month treatments, the average of P was derived from individual time intervals within the 30-day period and used to calculate doses. For 8-month treatments, a dose from the first month was summed with the dose from the subsequent 7 months. The latter was calculated using P measured at 30 days of exposure. Resulting internal β-particle dose rate and cumulative tritium dose (*i.e.* total dose accumulated during 1 and 8-month tritium treatments) estimates are presented in Table 2.

### Low dose chronic γ-irradiation

The Gamma Beam Irradiation Facility of the Biological Research Facility, equipped with an open beam (GammaBeam-150C, Atomic Energy of Canada Limited) was used for the low-dose γ-irradiations, with similar dose rate of tritium exposure (Table 2).

### M-FISH analysis

Mouse PBLs were isolated and cultured using techniques previously described [36]. Briefly, ACK lysis buffer (Life Technologies, USA) was used to isolate lymphocytes from peripheral blood collected by intra-cardiac venipuncture. Isolated lymphocytes were cultured in the presence of phytohemagglutinin (Life Technologies, USA) and lipopolysaccharide for 42 h. Colcemid (Life Technologies, USA) was added 18 h prior to harvesting and fixing cells for M-FISH staining using the “21× Mouse” probe kit (MetaSystems, Germany) as per manufacturer's recommendations. Between 200 and 500 metaphases from at least 2 mice per group were imaged and analyzed (Table 3). Chromosome aberrations were quantified and converted into chromosome breakpoints allowing us to evaluate chromosome damage rates as described in Supplemental Materials (Supplementary Figure 1).

**Table 3:** Lymphocyte metaphases analyzed by M-FISH in mice exposed to low doses of tritium in drinking water and to external γ-irradiation for 1 or 8 months

Treatment group	No of mice	No of metaphases	No of chromosome breakpoints
1 month			
Control	6	509	9
HTO 10 kBq/L	3	254	4
HTO 1 MBq/L	3	260	9
HTO 20 MBq/L	2	206	8
OBT 10 kBq/L	3	259	6
OBT 1 MBq/L	5	586	16
OBT 20 MBq/L	4	252	10
Gamma 1.44 μGy/h	3	268	7
Gamma 31.0 μGy/h	3	248	2
8 months			
Control	4	408	5
HTO 10 kBq/L	2	301	1
HTO 1 MBq/L	2	382	0
HTO 20 MBq/L	5	533	7
OBT 10 kBq/L	3	299	6
OBT 1 MBq/L	6	475	15
OBT 20 MBq/L	4	466	20
Gamma 1.44 μGy/h	4	403	0
Gamma 31.0 μGy/h	NA	NA	NA

NA: Not available.

### Micronucleus assay

The bone marrow micronucleus assay was conducted using a previously published protocol [31]. Briefly, two slides were prepared from each animal, fixed in methanol and stained with Acridine Orange. Frequencies of polychromatic erythrocytes (PCE) containing micronuclei (mn-PCE), as well as polychromatic to normochromatic erythrocyte ratio (PCE/NCE), were determined using fluorescence microscopy.

### Analysis of the dose-response for obt induced chromosome damage

The rates of chromosome damage after low doses and dose rates of OBT exposure (from both 1- and 8-month experiments) were modeled using a quasi-Poisson regression including a logarithmic dose-response relationship. In particular; if *Y* denotes a Poisson random variable counting the chromosome damage in a cell sample size *n*, then, we allow its mean *E(Y)* to be:

linear E(Y)=μ=c×n×b×n×Dose (1) or logarithmic E(Y)=μ=c×n+b×n×log(1+a×Dose) (2),

where *a*, *b* and *c* are the model parameters estimated by maximizing the Poisson Likelihood. The standard errors were computed using a quasi-Poisson method by incorporating the over-dispersion factor [64]. In order to reduce the number of estimated parameters due to the small sample size, we constrained the *c* parameter to be equal to the baseline (non-irradiated) chromosome damage rate. The significance of the dose-dependent terms in the models (1) and (2) was investigated using a permutation test [65, 66]. We compared the goodness of fit on the proposed model (2) vs. the linear model using the Akaike information criteria (AIC) [67], which penalizes the likelihood of the models by their numbers of explanatory variables in order to obtain the most parsimonious one. Lower AIC values indicate better fit and differences in AIC above 4 may be considered as significant. We carried out all calculations for the dose-response analysis with MATLAB Version: 8.2.0.701 (R2013b).

### Relative biological effectiveness (RBE) evaluation for induced chromosome damage

RBE was calculated as follows:

RBE_q_ = D_r_/D_q_,

where RBE_q_ is the relative biological effectiveness of type q radiation (here, OBT chronic exposure), D_r_ is the dose of the reference radiation (here, acute γ-irradiation) that produces the biological effect (here, chromosome damage rate) which is equal to that produced by a dose D_q_ of the type q radiation. For each step value of chromosome damage rate of 0.0001, the ratio of doses was calculated allowing us to plot the RBE curve. The 95 percent pointwise confidence intervals curves of the fitted RBE curve were obtained by bootstrapping [68]. The RBE values of OBT were estimated by calculating the ratio of doses only for the OBT-induced chromosome damage rate values significantly higher than the control chromosome damage rate value (0.0153 [0.0083 – 0.0256]).

### Statistical analyses

Differences in mn-PCE frequencies were evaluated using Student's *t*-test. The difference between the chromosome damage after tritium/irradiation vs. control was tested using a Poisson rate-ratio based test.

## CONCLUSIONS

Collectively, presented data suggest that tritium in the form of OBT may have a higher genotoxic potential compared to HTO and to external γ-radiation. In mouse PBLs, this genotoxic potential of very low doses of chronicle OBT exposure is higher than currently assumed. Furthermore, when compared to external acute γ-radiation, the estimated RBE value of OBT for chromosome aberration end-point highly varies in the dose range [1–200 mGy] of cumulative dose of OBT, with the highest values found for the lowest OBT doses. For doses of ~200 mGy the OBT decreased down to ~1. More accurate RBE evaluation would require the use of equivalent dose rate chronic γ-irradiation; however such γ-irradiation at equivalent to tritium doses did not result in cytogenetic damage. None of the treatments produced detectable cytogenetic damage in bone marrow suggesting a tissue specificity for tritium-induced cytogenetic damage. Importantly, 10 kBq/L did not exert genotoxicity in lymphocytes or bone marrow erythroblasts. Although presented data and estimates of RBE of OBT are of limited practical value, they highlight the need for further studies and such studies are underway. Current data will be considered and put into context with other end-points, such as physiological markers of toxicity and inflammation markers, DNA damage and repair, which will be measured in samples collected within this study, as well as data on tumorigenesis and life span, and will be reported in future publications as described in Gueguen *et al.* [15].

## SUPPLEMENTARY MATERIALS FIGURE



## Abbreviations

AIC: Akaike information criteria
DSB: double-strand break
HTO: tritiated water
LET: linear energy transfer
mn-PCE: polychromatic erythrocytes containing micronuclei
NCE: normochromatic erythrocyte
OBT: organically bound tritium
PBL: peripheral blood lymphocyte
PCE: polychromatic erythrocytes
RBE: relative biological effectiveness
