# Supplementary material for: Cytogenetic damage analysis in mice chronically exposed to low-dose internal tritium beta-particle radiation
Source: Oncotarget. 2018 Jun 8;9(44):27397–411. doi: 10.18632/oncotarget.25282 (PMC6007944; doi:10.18632/oncotarget.25282)
Supplement: Supplementary file 1 [file oncotarget-09-27397-s001.pdf]

Cytogenetic damage analysis in mice chronically exposed to low-dose internal tritium beta-particle radiation

SUPPLEMENTARY MATERIALS

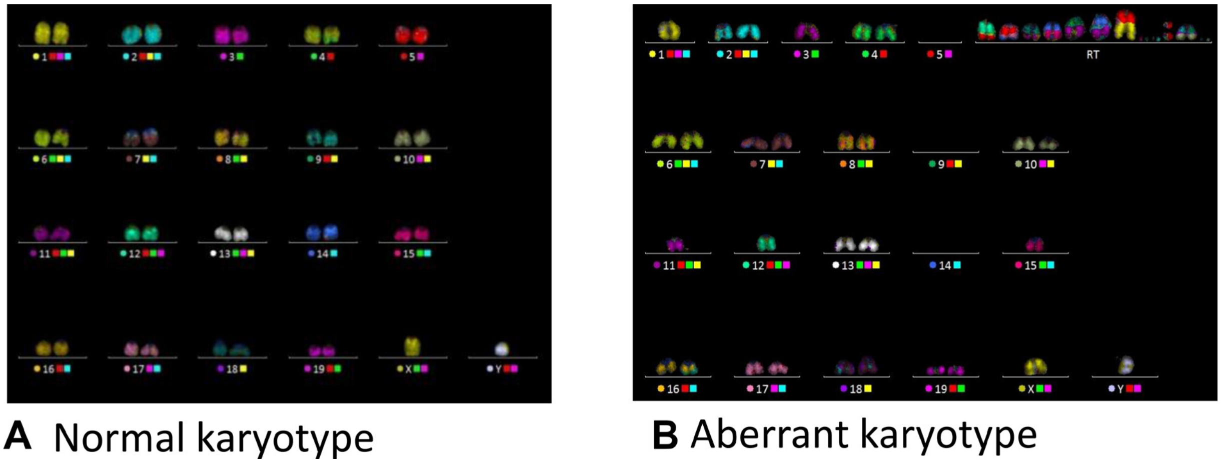

Figure 1: Mouse lymphocytes were processed using M-FISH as described in Materials and Methods and metaphase chromosomes were photographed and assembled together. (**A**) representative image of a normal karyotype. (**B**), example of an aberrant karyotype.
